# Dengue outbreak in the times of COVID-19 pandemic: Common myths associated with the dengue

**DOI:** 10.1016/j.amsu.2022.104535

**Published:** 2022-09-01

**Authors:** Saba Zaheer, Muhammad Junaid Tahir, Irfan Ullah, Ali Ahmed, Sheikh Mohd Saleem, Sheikh Shoib, Muhammad Sohaib Asghar

**Affiliations:** a Dow University of Health Sciences, Pakistan; b Lahore General Hospital, Lahore, 54000, Pakistan; c Kabir Medical College, Gandhara University, Peshawar, 25000, Pakistan; d School of Pharmacy, Monash University, Jalan Lagoon Selatan, Bandar Sunway, 47500, Subang Jaya, Selangor, Malaysia; e Independent Public Health Researcher, J&K, India; f Department of Psychiatry, Jawahar Lal Nehru Memorial Hospital, Srinagar, Kashmir, India; g Department of Internal Medicine, Dow University of Health Sciences-Ojha Campus, Karachi, Pakistan

**Keywords:** Dengue virus, Coronavirus, Myths, Attitudes, Knowledge

## Abstract

With the sharp rise in dengue cases across the state and the ongoing COVID-19 pandemic, it is crucial to pay attention to the common misbelieves among the population about dengue. It should be considered to actively spread awareness about the disease to bust the common myths associated with it. A few common myths include that it is a contagious disease, or it is a milder infection than COVID-19, so it's not taken more seriously, or that one cannot be coinfected with both dengue and COVID-19 at one time. We propose that accurate information about dengue can be spread through community education through televisions and social media to cater to the targeted audience. In addition to that, awareness campaigns in rural areas should be planned to help the masses understand the pathogenesis of the diseases and play a role in limiting the transmission.

Dengue cases are sharply rising across states and, in many places, even more, fearful than the coronavirus disease 2019 (COVID-19) threat right now [1]. In the past few decades, Dengue infection has spread rapidly within countries and across regions, resulting in an increased frequency of epidemics and severe dengue disease, hyper-endemicity of multiple dengue virus serotypes, putting nearly a third of the human population, worldwide, at risk of infection. More than 100 countries in tropical and subtropical areas demonstrated dengue endemicity, including Southeast Asia, Central, and South America, Africa, Western Pacific, and eastern Mediterranean regions [2]. The incidence of dengue has increased 30-fold in the last five decades with an accelerating geographical spread [3]. More than half of the human population lives in dengue-endemic areas. About 50 to 200 million dengue cases with 500,000 incidences of dengue haemorrhagic fever (DHF) and over 20,000 deaths are documented every year around the globe [4]. Moreover, under-reported infections are not well estimated for incidence and economic burden.

The disease also has a significant impact on the economy, a systematic review done in Latin America reveals that the main economic impact of dengue was due to its direct medical costs [5]. Average annual cost was more than US$ 3 billion. 70% of it was spent on hospitalized cases of dengue. For outpatients, direct medical costs were low, but social costs were significant [5].

Combating dengue comes with various challenges and hindrances in the path of eradication of disease within the region. One of the biggest challenges we face with the dengue-endemic is the deep-seated myths about the disease and its transmission amongst the local population. It should be considered to actively spread awareness about the disease to bust the common myths associated with it. The current COVID-19 pandemic has shown us the correct and effective way to spread information among the masses; therefore, it should be considered to use similar resources and ideas to let the accurate information about dengue infection reach the population so that we can fight this deadly endemic alongside COVID-19.

Dengue is a mosquito-borne disease. However, it is crucial to understand that there are many different types of mosquitoes, which can cause dengue infection. People believe that any mosquito bite can cause dengue. Still, the primary vector responsible for dengue transmission and dengue epidemic is *Aedes aegypti*, a species that belongs to the genus *Aedes*. Other mosquito species in the genus *Aedes* — including *Aedes albopictus*, *Aedes polynesiensis*, and *Aedes scutellaris* — can only serve as dengue vectors [6]. People tend to worry about being infected with dengue whenever they see a mosquito bite. Still, there are ways to differentiate whether the Aedes mosquito has bitten the person. The bite of the dengue-causing mosquito is much more starkly red and itchier than other mosquito bites. They tend to bite the most often during the daytime and bite below the knee, around the ankles, or elbows for most people. Another myth is that people often think that if one person in the family contracts the virus, it will also put everyone else at risk. This is far from the truth because dengue is not contagious, as it cannot be spread directly from person to person. However, a person infected and suffering from dengue fever can infect other mosquitoes, which can further infect other humans. Humans can carry the infection from one country to another or from one area to another during the stage when the virus circulates and reproduces in the blood system. But there is no direct transmission between humans [7].

Another prevalent myth among the masses is that the Aedes mosquito breeds only in dirty areas. At the same time, scientists have uncovered that Aedes mosquitos can lay their eggs in puddles as small as a twenty-cent coin. When it comes to dengue fever in Singapore, clean stagnant water in flower vases or pots, roof gutters, and bamboo pole holders are typical breeding areas. They may even lay their eggs in hardened soil pockets of water. To avoid breeding of Aedes mosquitos within our homes and nearby localities, the National Environment Agency has devised the acronym BLOCK. [B- break up hardened soil, L-lift, and empty flowerpot and plates, O-overturn pails and wipe their rims, C-change water in vases, K-keep root gutters clear and place insecticide]. In addition to this, keeping the house environment clean and regularly checking for any spots with stagnant water to keep mosquitoes from breeding are essential tools [8].

The other common assumption among people is that dengue is a milder infection than COVID-19. With the rapid spread of dengue in COVID-19, there is a fear of developing confusing symptoms and delaying the diagnosis and treatment. People tend to believe that, unlike COVID-19, dengue does not have fatal risks attached to it [9]. Although COVID-19 is a severe, deadly viral infection that has caused many deaths worldwide, dengue fever, also known as “Break-bone fever,” can be very dangerous and cause severe complications if not treated timely. Some also believe that they can get the dengue infection once in a lifetime. Unfortunately, the truth is busted and well revealed, and a person can contract dengue fever more than once. Dengue fever is divided into four serotypes of virus variants. As a result, even if a person acquires dengue and recovers, the immunity is limited to one serotype only with which the individual was infected.

Furthermore, people infected a second time may have worse symptoms than they did the first time. Ironically, scientists have discovered that when a person is reinfected with dengue fever, the immune response exacerbates the symptoms [10]. Like most infections, the severity of dengue infection can lie anywhere between be mild to severe. The chances of an infection turning into a severe one can significantly rise if treatment isn't sought in time. Some of the symptoms associated with a severe case of dengue include breathing difficulties, thrombocytopenia, acute hepatitis, and later stages progress to liver failure, delirium, and confusion. The newer variant, known as the DENV-2 serotype, is particularly being referenced to causing severe symptoms, high fever, and even lead to mortality in any cases. This variant can facilitate viral entry and cause the infamous DHF or dengue shock syndrome (DSS). This variant, DENV-2, is so concerning that it can spread fast and cause mortality if not managed timely. Moreover, this variant can be more threatening to individuals who have been previously infected by one dengue serotype and then get infected by this variant [9].

Once infected with dengue fever, apart from seeking medical treatment, many believe that dengue fever can be well treated by consuming papaya leaf juice. The medicinal benefits of papaya leaf juice for treating dengue fever are a common old wives' tale in Singapore. This is because when you have dengue fever, your platelet count drops dramatically. Platelets help our blood clot. Thus patients with a low platelet count are at a higher risk of internal bleeding. The juice of papaya leaves has been shown to help increase platelet count. There is, however, no scientific evidence that papaya leaf juice helps treat dengue fever [11]. In truth, there is no cure for dengue fever at the moment. Instead, doctors give medication to treat the symptoms of dengue fever. It's also essential to stay hydrated when it comes to healing from dengue fever. While most dengue fever occurrences are minor, getting treatment early might help you control the symptoms of the disease.

Another misconception that needs close attention is people believing they cannot be infected with COVID-19 and dengue simultaneously [12]. In Singapore, few cases have been reported where patients were initially tested negative for dengue but were hospitalized later due to persistent fever. The final diagnosis revealed co-infection with dengue and severe acute respiratory syndrome coronavirus 2 (SARS-CoV-2) [13]. Two dengue and COVID-19 co-infection cases have also been reported in Bangladesh, with death in one patient [9]. Another point of co-infection associated with death has been noted in India [9]. In Thailand, a patient who presented with petechial rashes was treated as a dengue case but declared co-infected with COVID-19 following advanced clinical diagnosis [14]. All of the above points prove that a person can be co-infected with both viruses, which carry the risk of fatality. People also tend to believe that only children and the elderly are at risk of contracting the dengue virus [9]. The aforementioned two age groups can be more susceptible to dangers because of their weak immune system; however, relatively healthier individuals are also vulnerable to contracting severe dengue infection. A study found that adults are more likely than young children to have clinical dengue [15].

Along with the rapidly rising dengue cases, these misbeliefs amongst people need close attention. There are many effective ways to control the transmission of disease but above all is controlling and changeling accurate information and awareness about the condition. Community education is one of the primary aspects that need to be enhanced promptly. This can be done by using television and social media to cater to a larger audience. There should be awareness campaigns in rural areas to help people understand the pathogenesis of this disease and how they can play a role in limiting the transmission. Masses should be encouraged for personal household protection measures, such as window screens, repellents, insecticide-treated materials, coils, and vaporizers. Wearing clothing that minimizes skin exposure to mosquitoes should be advised [16]. On a much higher level, the district and provincial authorities should develop Standard Operating Procedures (SOPs) of water management for timely cleaning of roads and places to avoid any stagnant water. The government should provide funds to authorities. The healthcare worker should become vigilant in differentiating the patients with confusing symptoms for COVID-19 and dengue. Moreover, these co-infected patients should be treated similarly for both COVID-19 and dengue. A brief literature has been added in Table 1 about Dengue control interventions.

**Table 1:** Dengue and control interventions.

S.no	Study	Description
1.	Gurevitz et al. [17]	This study reveals how social, physical, and biological processes shape dengue transmission and suggest multiple opportunities for control interventions.
2.	Kusuma et al. [18]	This study demonstrated health education based interventions increase knowledge, which is a prerequisite for changing/adopting certain protective behaviours. However, educating the communities is not enough and should be coupled with community based environmental management and effective vector control measures through community participation. It is important to strengthen the training component of the healthcare staff in health education and communication strategies to work with the communities.
3.	Soo et al. [19]	This study provides evidence that the presence of certain serotypes, including primary and secondary infection from South East Asia and Non-South East Asia regions, increased the risk of severe dengue infections. Thus, these serotypes are worthy of special consideration when making clinical predictions upon the severity of the infection.
4.	Cardona-Ospina et al. [20]	This study analyses the current epidemiological trends of dengue and COVID-19. As both conditions may potentially lead to fatal outcomes, especially in patients with chronic co‐morbidities, overlapping infections, and co‐occurrence may increase the number of patients requiring intensive care and mechanical ventilation.

**Recommendations and way forward:** Diagnosis and case management of dengue cases needs to be done as per the guidelines laid down by World Health Organisation. Integrated surveillance and outbreak preparedness of vector-borne disease need to be increased. The surveillance system for dengue should be part of the national; health information system, with a set of core indicators, monitored at various levels of health administration. Sustainable vector control. Vaccine implementation, basic operational and performance research in vector-borne diseases. Unique advocacy campaigns should target the public, private, and sectors involved in developing new products for dengue prevention and control.

## Data availability

All the data is available in the public domain and is freely available.

## Author contributions

M.J.T and S.Z conceived the idea, S.Z, I.U, M.J.T, S.S, A.A and S.M.S retrieved the data, did write up of the manuscript, and finally, S.S, A.A, S.M.S, M.S.A and I.U reviewed and provided inputs. All authors approved the final version of the manuscript.

## Financial support

No financial support was acquired for this article.

## Declaration of competing interest

The authors declare no conflict of interest.
